# Feline Coronavirus Prevalence in 493 Cats With Chronic Diarrhea

**DOI:** 10.1111/jvim.70103

**Published:** 2025-04-26

**Authors:** Gary D. Norsworthy, Kristin N. Miller, Sarah M. Castro, Diane D. Addie

**Affiliations:** ^1^ Alamo Feline Health Center San Antonio Texas USA; ^2^ Well Pets Veterinary Clinic and After‐Hours Urgent Care Irmo South Carolina USA; ^3^ Catvirus.com, Maison Zabal Etchebar France

**Keywords:** chronic enteropathy, *Clostridium perfringens*, diarrhea panel, *Giardia*, inflammatory bowel disease, *Tritrichomonas*

## Abstract

**Background:**

Chronic diarrhea (CD) is common in cats, with unknown etiology in many cases.

**Objectives:**

To establish the prevalence of feline coronavirus (FCoV) and other enteropathogens in cats with CD.

**Animals:**

Veterinarians at a feline specialty practice examined 493 cats with CD. The breed of three (0.6%) was unknown; 373 (75.7%) were non‐purebred, and 117 (23.7%) purebred.

**Methods:**

Retrospective database review of 586 fecal sample results of an RT‐PCR and PCR diarrheal panel.

**Results:**

Feline coronavirus was found in 321 (65.1%) of 493 cats with CD. FCoV RNA and *Clostridium perfringens* toxin DNA were detected in 377 (64.3%) and 319 (54.4%) samples respectively: 206 (35.2%) samples were positive for both pathogens. Feline coronavirus was the sole pathogen detected in 118 (23.9%) cats. Samples from 203 cats under 1 year old were significantly (*p* = 0.0001) more frequently FCoV positive than samples from older cats (166/224 [74.1%] samples vs. 211/362 [58.3%]). FCoV RT‐PCR positivity peaked in February (*p* = 0.016) and March (*p* = 0.0064). Other detected pathogens included *Giardia* spp. (8.4%; 49/586 samples); *Tritrichomonas blagburni* (8.4%; 46/586); *Cryptosporidium* (5.1%; 30/586); *Campylobacter jejuni* (3.4%; 17/497); *Campylobacter coli* (1.6%; 8/497); *Salmonella* spp. (0.8%; 5/586); panleukopenia virus (0.8%; 5/586); and *Toxoplasma gondii* (0.5%; 3/586). Sixty‐nine cats gave 162 samples: 54/69 (78.3%) cats were FCoV positive, 39/54 (72.2%) persistently so.

**Conclusions:**

If FCoV is non‐pathogenic, as often assumed, its having the highest rate of positivity in CD cases is difficult to explain. If pathogenic and overlooked, key diagnostic and therapeutic opportunities might be missed.

**Abbreviations**
CD: chronic diarrhea
FCoV: feline coronavirus
IBD: inflammatory bowel disease
*n*
: number
PCR: polymerase chain reaction
RT‐PCR: reverse‐transcriptase polymerase chain reaction

## 1 Introduction

Chronic diarrhea (CD) is defined as feces with a barely‐formed to watery consistency that is present for at least 3 weeks [1, 2]. It is a major problem in cats, accounting for 8.5% of feline visits to British veterinary practices [3], and diarrhea accounts for 1.8% of 15,226 cat visits in the USA [4].

The causes are diverse: including infections, parasites, diet‐responsive enteropathy, neoplasia, and extra‐intestinal causes (e.g., hepatic, renal or pancreatic disease [1]). Many cats are left with a diagnosis of inflammatory bowel disease (IBD) [1, 5, 6, 7] which is a diagnosis of exclusion, and its underlying pathogenesis is not fully understood.

FCoV is a recognized cause of acute diarrhea [8], usually occurring in kittens [9, 10], but it can affect cats of any age and can be fatal [11]. Chronic FCoV infection can cause chronic large‐bowel diarrhea [12], sometimes with fecal incontinence. FCoV is frequently found in the feces of diarrheic cats [8, 13, 14, 15], but it is also found in the feces of cats without diarrhea [16]; therefore, its presence in a diarrhea panel is often ignored.

The role of intestinal bacteria in cats with chronic enteropathy has received much recent attention [2, 6, 17, 18], whereas that of intestinal viruses has not. Our aim for this paper was to retrospectively examine 13.3 years of records of a feline specialty hospital to establish the prevalence of FCoV infection among cats with CD in North America.

## 2 Materials and Methods

### 2.1 Data Collection

We retrospectively reviewed the clinical records of 493 cats that were presented to Alamo Feline Health Center in San Antonio, Texas, USA between December 29, 2008 and May 30, 2022. Each cat had diarrhea for at least 3 weeks, many for several months, had a negative fecal flotation exam, and had undergone various treatments, including drugs, supplements, and therapeutic diets.

As part of the standard diagnostic workup, owners were instructed to collect feces within 24 h of defecation. A total of 586 feline fecal samples were submitted to a commercial veterinary reference laboratory^1^ for a real‐time PCR assay evaluating a panel of 10 enteropathogens as previously described [19], including detection of the *Clostridium perfringens* enterotoxin gene as well as the alpha‐toxin gene. The diarrhea panel also tested for FCoV RNA and the DNA of *Giardia* species (spp.); *Tritrichomonas blagburni* (*T. blagburni* formerly called *T. foetus*); *Cryptosporidium* spp.; *Campylobacter jejuni* ; *Campylobacter coli* ; *Salmonella* spp.; panleukopenia virus; and *Toxoplasma gondii*.

### 2.2 Statistical Analysis

Statistical analysis of seasonal variation was by Poisson Probability Distribution with *p* value set at < 0.01. The FCoV prevalence in domestic versus purebred cats, and in cats less than or at least 1 year of age was examined using the Fisher exact test with significance set at < 0.05.

## 3 Results

### 3.1 Cats

The breeds of 490 out of 493 cats were recorded; 373 (76%) were domestic short‐, medium‐, or long‐haired cats, and 117 (24%) were purebred, including 14 purebred crosses (Table 1). The gender of the cats is summarized in Table 1; 185 (86.8%) of 213 female cats were neutered, and 257 (91.8%) of 280 male cats were neutered.

**TABLE 1:** The breeds of cats presented for chronic diarrhea, their sex, age, and FCoV fecal RNA status.

	Number of cats	F	Fn	M	Mn	Number of kittens < 1 yo	Total FCoV positive (%)	< 1 yo FCoV positive (% of kittens)
Domestic (short, medium or long hair)	373	21	139	15	198	142	243 (65.1)	103 (72.5)
Purebred	117	6	46	8	57	61	77 (65.8)	47 (77.0)
Unspecified/other	3	1	0	0	2	0	1 (33.3)	0 (0)
Total	**493**	**28**	**185**	**23**	**257**	**203**	**321**	**150**

*Note:* The table above shows whether cats whose fecal samples were tested were domestic or purebred; their sex; whether their feces tested positive for FCoV RNA; the percentage of domestic and purebred cats which were FCoV positive; the number of kittens under 1 year of age, the number of FCoV positive kittens under 1 year of age, and the percentage of kittens which were FCoV‐positive.

Abbreviations: F: female intact; FCoV: feline coronavirus; Fn: female neuter; M: male intact; Mn: male neuter; yo, year old.

The median age of cats at the time of testing all 586 samples was 2.03 years (range of 16 days to 19.8 years). Two hundred and twenty‐four (38.2%) of the 586 samples were from 203 cats under 1 year of age.

### 3.2 Detection of Infections

**FIGURE 1:**
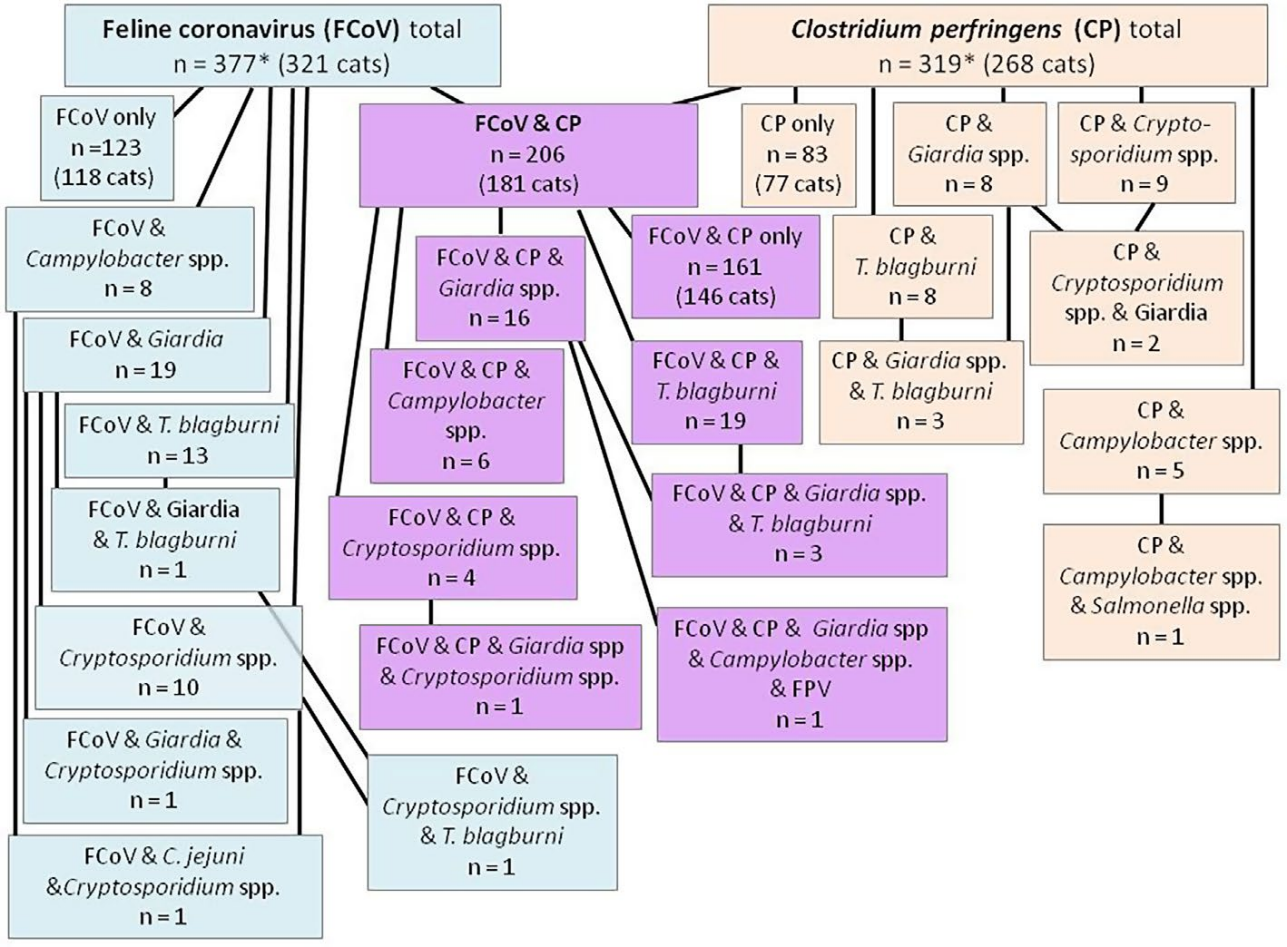
Co‐infections involving feline coronavirus and *Clostridium perfringens*. This figure shows a breakdown of the numbers of samples co‐infected with FCoV and *C. perfringens* , or *C. perfringens* . *Only the major co‐infections of FCoV and CP are shown: Space did not permit showing tests where only one co‐infecting pathogen was positive. A complete list of all positive test results is given in Table 2. FCoV: feline coronavirus; *n*: number; *T. blagburni*: *Tritrichomonas blagburni* (formerly called *T. foetus*).

The number of samples positive for each infection detected is shown in Table 2. The most frequently detected pathogens were FCoV and *C. perfringens* , with 377 (64.3%) and 319 (54.4%) positive samples respectively; 206 (35.1%) samples were positive for both FCoV and *C. perfringens* (Figure 1). FCoV and *C. perfringens* infections were the sole pathogens detected in 21.0% and 14.2% of samples, comprising 32.6% and 26.0% of all FCoV and *C. perfringens* infections. Sixty (10.2%) samples from 54 cats tested positive for at least three co‐infections, and 45 of those samples (from 42 cats) contained both FCoV and *C. perfringens* . Nine samples contained FCoV without *C. perfringens* , and six *C. perfringens* without FCoV. Only one double‐infected sample had a co‐infection not involving FCoV or *C. perfringens* ; that cat was co‐infected with *T. blagburni* and *Giardia* spp.

**TABLE 2:** Overview of 586 IDEXX diarrhea panel PCR test results from cats presented to Alamo Feline Health Center.

Organism	Samples positive (% of total 586 samples)	How many cats infected (% of total 493 cats tested)	Samples with a single infection
Feline coronavirus	377 (64.3%)	321 (65.1%)	123 (21.0%)
*Clostridium perfringens*	319 (54.4%)	268 (54.4%)	83 (14.2%)
*Cryptosporidium* spp.	30 (5.1%)	26 (5.3%)	7
*Giardia* spp.	49 (8.4%)	42 (8.5%)	3
*Tritrichomonas blagburni*	46 (7.8%)	40 (8.1%)	2
*Campylobacter jejuni*	17^a^ (3.4%)	17 (3.4%)	0
*Campylobacter coli*	8^a^ (1.6%)	7 (1.4%)	2
*Salmonella* spp.	5 (0.9%)	5 (1.0%)	0
*Panleukopenia*	5 (0.9%)	5 (1.0%)	0
*Toxoplasma gondii*	3 (0.5%)	3 (0.6%)	0

*Note:* This table shows the number of samples and cats positive for each infection on the diarrhea panel.

^a^ Only 497 samples were tested for *Campylobacter* spp.

Eighty‐one (13.8%) samples from 79 cats tested negative for all infections. Two cats tested negative for all pathogens on two separate occasions. Ten of the 81 completely negative samples were repeat samples, eight of which were taken to confirm the effectiveness of treatment for a specific pathogen.

### 3.3 Feline Coronavirus

FCoV was the most prevalent pathogen found in this cohort of cats; 377 (64.3%) of 586 samples were positive; 321 (65.1%) of 493 cats were infected. FCoV was the only enteropathogen identified in 123 (21.0%) samples from 118 (23.9%) cats. The prevalence of FCoV is shown in Tables 1 and 2, Table S1, and co‐infections are shown in Figure 1.

#### 3.3.1 Breed of FCoV‐Infected Cats

Of 373 non‐purebred cats tested, 243 (65.1%) were positive for FCoV RNA on at least one test, which was not significantly different from 77 (65.8%) of 117 purebred cats (*p* = 0.91) which were FCoV infected. Of the three cats with unspecified breed, one tested positive for FCoV RNA.

#### 3.3.2 Age of FCoV‐Infected Cats

**FIGURE 2:**
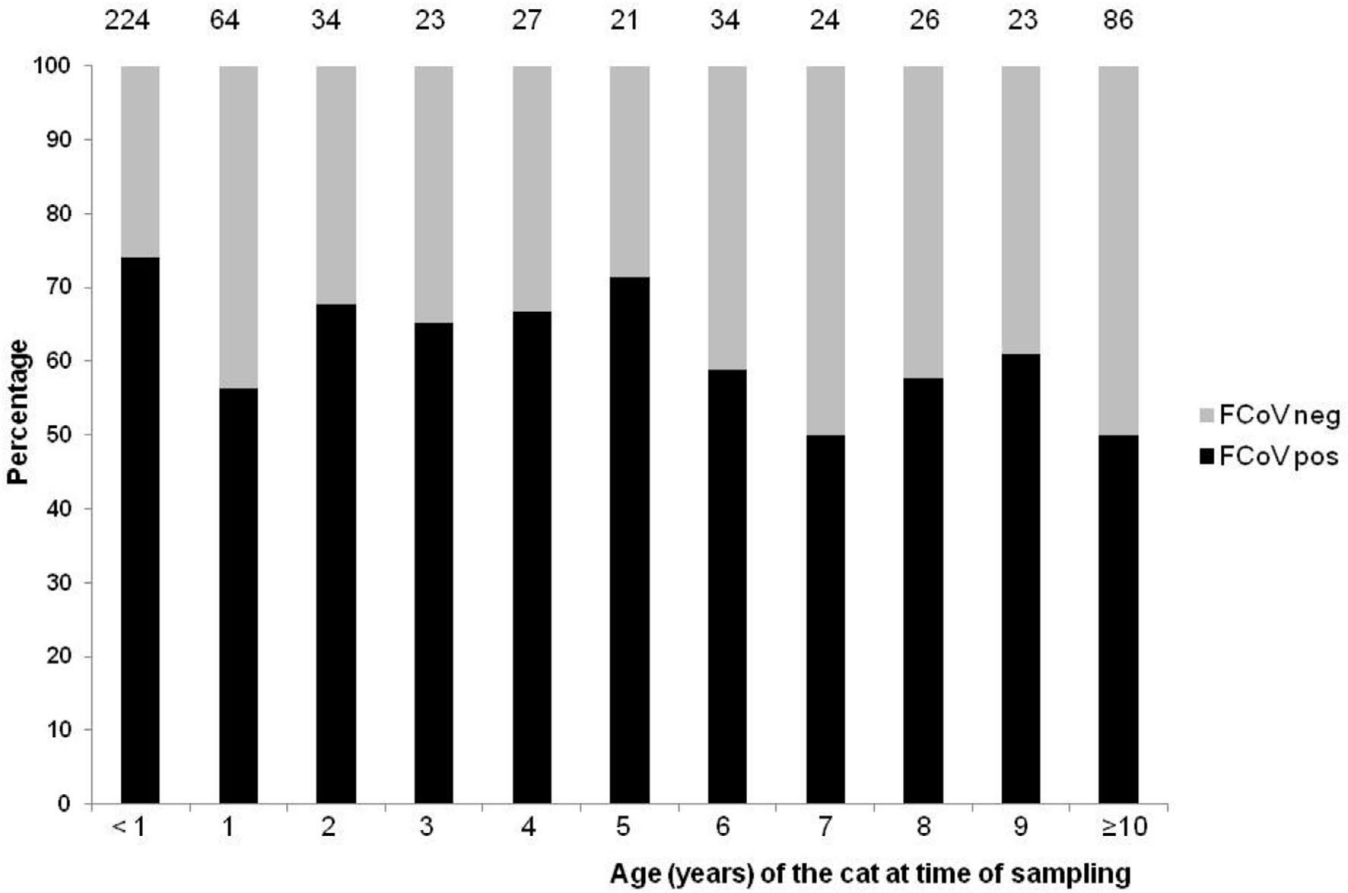
The percentage of samples positive for FCoV RNA at various cat ages. This bar chart shows the percentage of samples positive for FCoV RNA at each age of the cats (in years). The numbers of samples tested at each age are given at the tops of the columns. Kittens under 1 year of age had the highest prevalence of FCoV at 74.1%, compared with 58.3% of samples from cats aged at least 1 year being positive.

The percentage of cats positive for FCoV at each age is shown in Figure 2. Kittens under 1 year of age were significantly more likely to be FCoV positive than adult cats; 166 (74.1%) of 224 samples from cats under 1 year old were FCoV positive compared with 211/362 (58.3%) samples from cats at least 1 year of age (*p* = 0.0001). The median age of cats when they were detected as being FCoV infected was 1.5 years (range 47 days to 17.6 years), which was younger than the median age of 4.0 years of the FCoV negative cats (range 16 days to 19.8 years).

#### 3.3.3 FCoV Prevalence Was Highest in February and March

**FIGURE 3:**
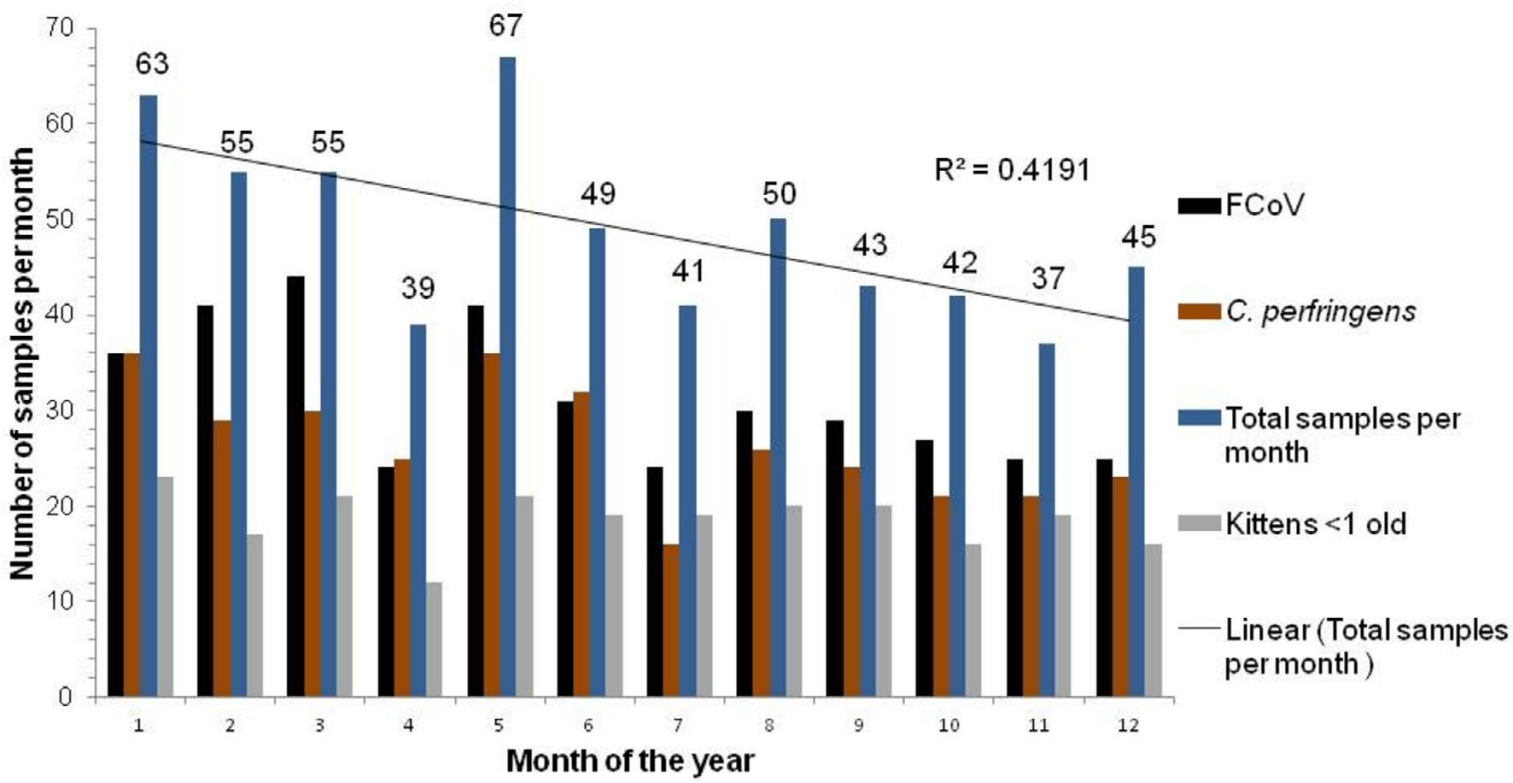
Total number of diarrhea panel tests in each month of the year (over 13.3 years). This bar chart shows the total number of samples sent for a diarrhea panel in each month of the year in the blue columns, with samples from cats under 1 year of age in gray. The trendline shows a weak tendency for sample numbers submitted for all tests to fall towards the end of the year (*R* ^2^ = 0.4191). FCoV positive samples (black columns) rose from January, peaking in February and March (*p* = 0.016 and *p* = 0.0064 respectively), then declined towards the end of the year. The total number of samples positive for *Clostridium perfringens* is shown in brown.

A seasonal trend was observed in both FCoV prevalence and the number of tests performed, with test numbers declining towards November (Figure 3), although the association was weak (*R* ^2^ = 0.4191; i.e., only 41.9% of diarrhea was associated with the month of the year). The number of samples submitted for testing peaked in January (*n* = 63) and May (*n* = 67) and was significantly more (*p* = 0.007 and *p* = 0.003 respectively) than the mean of 49 (i.e., 3.6 samples per month per year).

**FIGURE 4:**
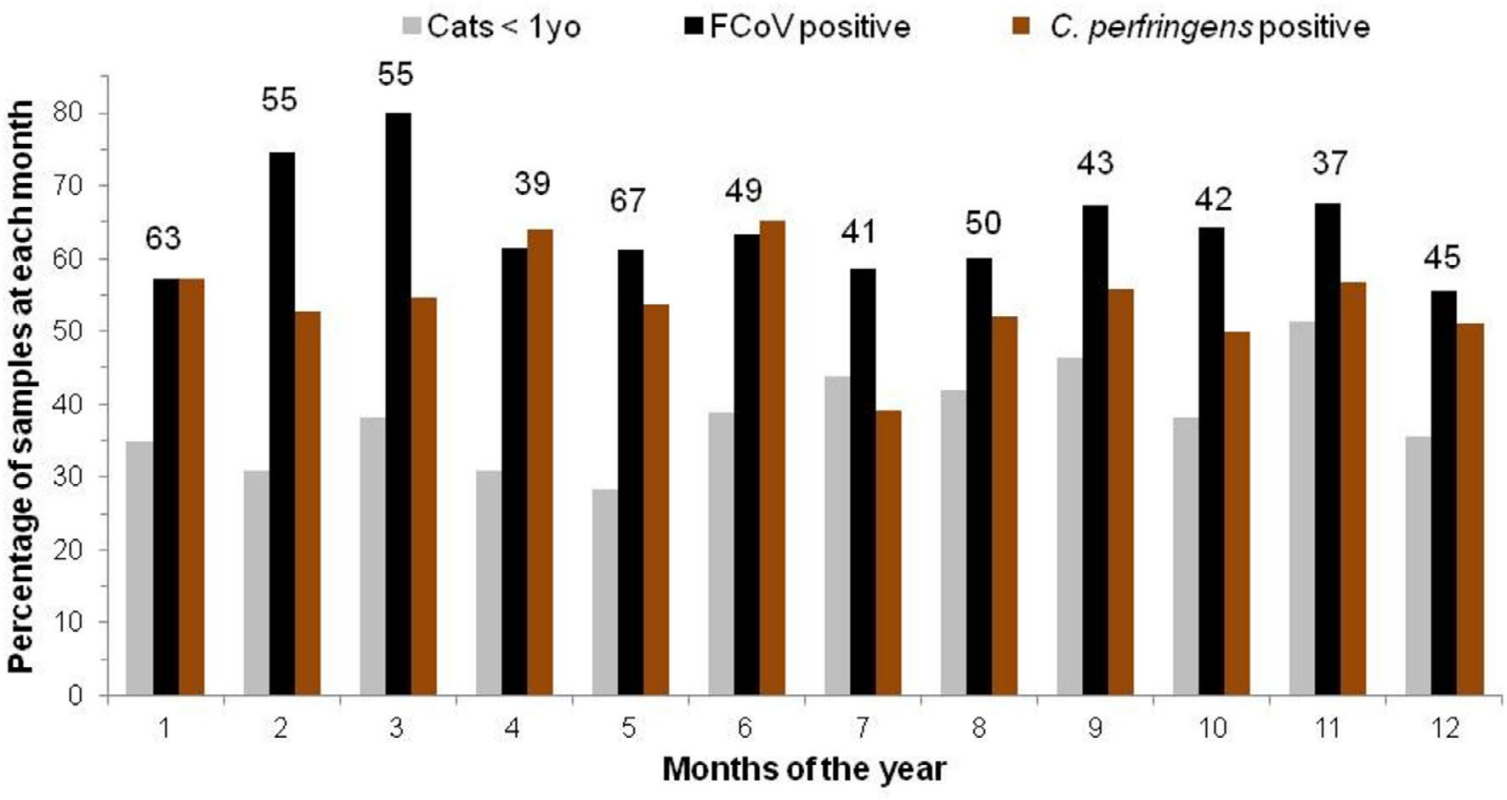
Percentage of tests positive for FCoV and *Clostridium perfringens* in each month of the year, showing the percentage of samples that were from cats under 1 year old. The total number of samples submitted for each month of the 13.3 years of the study is shown for each month at the top of the blue columns. The percentage of samples positive for FCoV (black columns) was highest in February and March. The FCoV peak was not due to a peak of kittens presented (shown in the gray columns) because the percentage of samples from cats under 1 year old presented each month peaked from summer (northern hemisphere) until November. The percentage of samples positive for *C. perfringens* is shown in brown: No apparent seasonal variation was evident.

The percentage of samples positive for FCoV RNA peaked significantly in February (74.5%, 41/55) and March (80%, 44/55; *p* = 0.016 and *p* = 0.0064 respectively). To examine whether this increase in FCoV was due to the tested population containing a larger proportion of kittens (which were more likely to be infected with FCoV than adult cats), the percentage of samples which were from cats under 1 year of age is shown in Figure 4. The peak of FCoV RT‐PCR positivity noted in February and March was not associated with a younger population being presented in those months; kittens formed a higher proportion of presented cats from summer onwards.

### 3.4 C. perfringens

*C. perfringens* was the second most prevalent pathogen and was detected in 268 cats; 319 (54.4%) of 586 samples were positive. Two hundred six (64.6%) of the 319 *C. perfringens‐positive* samples were co‐infected with FCoV (Table 2, Figure 1). *C. perfringens* was the sole pathogen in 77 cats (83/319 (26.0%) *C. perfringens* positive samples).

### 3.5 Sequential Tests of 69 Cats

#### 3.5.1 Re‐Tested Cats

One hundred and sixty‐two samples were from 69 cats; 93 samples were re‐tested, and 54 cats were re‐tested once only. The median interval between the first and last test was 4.7 months (range 11 days to 4.7 years). Fifty‐four of the 69 re‐tested cats were non‐purebred (78.3%), and 15 (21.7%) were purebred. Purebred cats were not more likely than non‐purebred cats to be re‐tested (*p* = 0.76).

Table 3 breaks down which enteropathogens were detected in this cohort of cats, and the columns indicate whether those infections were detected on every test, were eliminated, acquired, or detected intermittently.

**TABLE 3:** Rates of Infection in 69 cats that were re‐tested.

Microbe	Number of infected cats retested	Cats positive every test (%)	Cats became negative (%)	Cats became positive (%)	Intermittent RNA/DNA detection (%)
Feline coronavirus	54	39 (72.2%)	7 (13%)	5 (9.3%)	3 (5.5%)
*Clostridium perfringens*	52	27 (51.9%)	14 (26.9%)	7 (13.5%)	4 (7.7%)
*Cryptosporidium* spp.^a^	11	1 (8.3%)	8 (66.7%)	2^a^ (16.7%)	0
*Giardia* spp.	10	2 (20%)	7 (70%)	1 (10%)	0
*Tritrichomonas* spp.	11	0	9 (81.8%)	1 (9.1%)	1 (positive in 6/8 tests)
*Campylobacter jejuni*	6	0	4	2	0
*Campylobacter coli*	3	1	1	1	0
*Salmonella* spp.	2	0	2	0	0
Panleukopenia	0	0	0	0	0
*Toxoplasma gondii*	0	0	0	0	0

*Note:* The cats becoming negative column shown above does not take into account cats becoming negative due to treatment (apart from FCoV, which was not being treated during the study period because no antiviral was available until after the study period ended). This table shows the outcome of infections amongst 69 cats whose fecal samples were tested more than once.

^a^ In addition to the two cats that became infected after testing negative, the 2nd of 8 samples from one cat gave a dubious positive Cryptosporidium PCR result, which we suspect to have been a false positive result. Therefore, the total number of positive Cryptosporidium‐infected samples might have been as high as 12.

#### 3.5.2 FCoV Infection Outcome

Fifty‐four (78.3%) of 69 cats were FCoV positive, and 15 cats were FCoV‐negative at every test (2 or 3 tests). Seven of the 54 positive cats became negative, and five cats tested negative on the first test and then tested positive (Table 3). Thirty‐nine (72.2%) of 54 cats were positive for FCoV on all two to five tests. Three more cats had a mix of positive and negative tests, including one cat that was tested eight times over 4 years, with six of the eight samples testing FCoV positive.

The median age of seven cats that eliminated FCoV was 4.9 years (range 4.0 months to 11.7 years). The median age of cats repeatedly positive for FCoV RNA was 2.0 years (range 3.2 months to 15.5 years). The appearance of older cats being more likely to clear FCoV infection than younger cats was not statistically significant (*p* = 1.0), possibly due to the low number of cats that became negative.

Excluding two outliers which were not re‐tested until over 1 and 2 years after their first test, the median time for five cats to become FCoV negative was 2.3 (range 0.7–5.6) months. The median time over which cats that tested positive on consecutive FCoV tests were monitored was 3.8 months (range 15 days to 4.6 years). Previous research defined FCoV carrier cats as those that consistently tested positive for FCoV RNA in their feces for at least 9 months [12]. Seventeen (31.5%) of the 54 FCoV‐infected cats for which we had repeat tests were positive for over 9 months, and one other cat was a probable carrier, being only 9 days short of a 9‐month interval at her last FCoV positive test.

To determine whether co‐infection with *C. perfringens* affected a cat's ability to recover from FCoV, we compared rates of positivity: 21 of 39 (53.8%) cats that stayed FCoV RNA positive on each test were also positive for *C. perfringens* toxins, compared with three of seven (42.9%) cats that became FCoV negative: the difference was not significant (*p* = 0.69).

#### 3.5.3 *C. perfringens* Infection Outcome

Fifty‐two of 69 (75.4%) re‐tested cats (94 samples) tested positive for *C. perfringens* enterotoxin genes, and 17 (24.6%) cats were negative at every test (2–3 tests). Twenty‐seven of 52 cats (51.9%) were positive on every test (2–5 tests; Table 3). Seven (13.5%) cats tested negative on the first test and then tested positive, and enterotoxin genes were intermittently detected in 4 (7.7%) cats. Fourteen positive cats became negative for *C. perfringens* enterotoxin genes, and of those, three became negative for FCoV, and one cat each became negative for Giardia, Salmonella, and Campylobacter.

One cat remained positive for FCoV and *C. perfringens* for at least 4.6 years and later became infected with *Giardia* spp., remaining positive for 3.7 years. The cat eventually tested negative for *Giardia* spp. on the fifth test following treatment with fenbendazole, followed by metronidazole (she had not recovered 3 years earlier when treated with metronidazole alone).

## 4 Discussion

We present a detailed description of the prevalence of FCoV in a large cohort of cats with chronic diarrhea with a close examination of co‐infections, especially *C. perfringens* , and seasonal presentation.

The primary limitation of this study is the lack of a historical control group of cats without CD, as well as the absence of a cohort of cats with acute diarrhea. However, performing a diarrhea panel on cats with normal bowel movements or those that recovered within a few days, whether or not they received anti‐diarrheal treatment or enteric friendly diets, would not be justified. Therefore, the only option remaining is a comparison of our FCoV prevalence with those of other studies. The 65.1% prevalence of FCoV RNA in the feces of this cohort of pet cats was similar to those of other studies where feces from cats with diarrhea were tested [13, 14, 15] but higher than 29% found in Korea [8] and considerably higher than 16%–36% in those studies where feces from cats that appeared to be healthy were tested [13, 20, 21, 22] and that were not in a multicat environment, where FCoV tends to be endemic. In a study of German breeding catteries, FCoV was found in 87% of 23 cats with diarrhea, compared with 59% of 211 cats without diarrhea [16]. The authors concluded that FCoV “can be associated with diarrhea.” [16] Association does not equal causation. However, in a kind of reversal of Koch's postulates, if a FCoV‐infected cat with CD recovers after administration of an antiviral drug specific for coronavirus, then it is reasonable to conclude that FCoV was the cause of the CD, and such was the finding of one group [23]. That said, FCoV clearly does not cause diarrhea in every cat it infects, and the proportion of FCoV‐infected cats that develops diarrhea is unknown. Nor is it known why a cat which has had normal stools for months or years despite FCoV infection suddenly develops diarrhea, which is responsive to an oral (but not injectable) anti‐FCoV drug (GS‐441524) [23]. However, FCoV is often present when some other condition is the true cause of the cat's diarrhea: for example the prevalence of low‐grade intestinal T‐cell lymphoma was 41.3% in our previous study of 300 cats with chronic gastrointestinal disease [5], and many of those cats were FCoV positive.

Our study revealed a peak of FCoV RT‐PCR positivity in February and March, differing from a large study of diarrheic Korean cats which found a seasonal peak of viral infections in November and of bacterial infections in October, but in that study their November and October peaks did not include FCoV and *C. perfringens* , which were found at high rates throughout the year [8].

Persistently infected FCoV carrier cats are prone to CD and, eventually, fecal incontinence [12, 24]. Other than the conservation of the strain of FCoV being shed [24], the only other marker currently to differentiate FCoV carrier cats from transient virus shedders is persistent fecal viral RNA positivity for 9 months or longer [12]. We had repeat samples for 69 cats, and 54 of those were infected with FCoV. Only seven (13%) cats became FCoV negative, which was considerably fewer than 56 of 141 (39.7%) naturally infected cats that stopped shedding virus in a previous study of subclinically infected cats [12]. Thirty‐nine (72.2%) of 54 FCoV‐infected cats were positive for FCoV RNA on all tests, and the percentage of persistently infected (i.e., carrier cats testing positive for at least 9 months) in our cohort of diarrheic cats was 31.5% (17/54); this was much higher than 13% found in the older study of subclinically infected cats [12], suggesting that the FCoV carrier status was responsible for clinical signs of enteropathy. The percentage of carrier cats in our study was likely underestimated because follow‐up testing of persistently FCoV‐positive cats was under 9 months in 22 cats.

We were unable to determine whether intermittent FCoV RT‐PCR positivity in the five cats that became infected and the three with mixed positive/negative results was due to intermittent viral shedding or re‐infection. Unfortunately, the hospital database did not include the number of cats per household, preventing us from assessing the likelihood of re‐infection.

The occurrence of intermittent viral shedding remains a subject of debate. Initially proposed by Addie and Jarrett in 2001 [12], it was later reconsidered by Addie in light of subsequent evidence. In 2023 [23], she postulated that what appears to be intermittent shedding may instead be attributable to re‐infection (typically via shared litter trays in multi‐cat households), PCR inhibition by fecal inhibitors [25], interference from cat litter leading to false‐negative results, or viral shedding occurring at the assay's detection limit, which could also account for false‐negative results [23].

Another limitation of our study was the inability to obtain cycle threshold (*C*_*T*_) values from IDEXX Laboratory, preventing us from determining whether cats that appeared to shed the virus intermittently were actually shedding very low levels of FCoV at the assay's limit of detection. Additionally, the laboratory does not report PCR inhibition, and since some samples were submitted with cat litter attached, the possibility of PCR inhibition leading to false‐negative results cannot be ruled out. It is also important to note that most cats with repeat samples were re‐tested only once. Given these limitations, the reported outcomes for FCoV‐infected cats in this cohort should be interpreted with caution. However, to our knowledge, this study is the only investigation of FCoV in diarrheic cats that includes any follow‐up data.

Intermittent shedding or a cat testing positive for *C. perfringens* toxin genes is expected, as it is a normal gut commensal that sporadically produces enterotoxins. However, it is more challenging to explain why some diarrheic cats appeared to acquire other new infections upon re‐sampling unless they originated from a multicat household where transmission from another cat was possible.

Although Giardia PCR is reported to be more sensitive than the antigen test [26], we have observed four cases in the past 2 years where samples tested antigen positive but PCR negative. This suggests that insensitivity, resulting in a false negative initial test, could explain cases where a cat initially tested negative for *Giardia* but later tested positive. Additionally, we observed unexpected positive results for *Cryptosporidium* and *Tritrichomonas* spp., raising the question of whether the initial test yielded a false negative result or whether subsequent positive tests were false positives in cases where new exposure to infection was unlikely.

In cats infected with multiple pathogens, only one cat tested negative for both FCoV and *C. perfringens* , raising the question of whether these two pathogens contribute to immunosuppression, thereby facilitating co‐infections, or if they act synergistically in causing diarrhea. We are not the first to observe a potential synergy between FCoV and *C. perfringens* [27]. Our findings align with a previous study that reported a higher likelihood of *T. blagburni*‐infected cats being co‐infected with FCoV and *C. perfringens* , or *C. perfringens* [14]. In our study, 31 of 41 *T. blagburni*‐positive samples were co‐infected with FCoV, 27 with *C. perfringens* , and 17 with both.

In FCoV carrier cats, the virus replicates in the large intestine [28]. FCoV‐infected cells release a substance which results in apoptosis of local lymphocytes [29], presumably preventing destruction of FCoV‐infected cells by natural killer cells and allowing FCoV infection to persist. This substance was later shown to be TNF‐alpha [30]. Amongst the cohort of cats presented in this study, we did not have enough sequential samples from cats who eliminated FCoV infection to analyze whether clearance of FCoV was more likely to occur in cats that were *C. perfringens* negative than positive. However, we observed a tendency for the converse to occur: a reduction of *C. perfringens* toxin production seemed to be associated with elimination of enteropathogens such as FCoV, *Giardia* spp., *Salmonella* spp., and *Campylobacter* spp.

The two main enteropathogens detected in this study are also commonly found in animals without diarrhea. FCoV is often regarded as a nonpathogen. If this assumption is correct, its presence in 65% of cats with CD remains unexplained. However, if FCoV does play a pathogenic role and its presence is overlooked, we risk missing significant diagnostic and therapeutic opportunities.

## 5 Conclusion

A retrospective analysis of a PCR‐based diarrhea panel for 10 enteropathogens, performed on 586 fecal samples from 493 cats with chronic diarrhea, revealed that FCoV RNA was detected in 321 (65.1%) cats. *C. perfringens* was the second most prevalent pathogen, with either its alpha‐ or enterotoxin DNA (or both), identified in 268 (54.4%) cats. The remaining eight pathogens were each detected in fewer than 10% of cases.

## Disclosure

The authors declare no off‐label use of antimicrobials.

## Ethics Statement

The authors declare no Institutional Animal Care and Use Committee or other approval was needed. The authors declare human ethics approval was not needed.

## Conflicts of Interest

Diane D. Addie is a member of the European Advisory Board of Cat Disease with no conflicts of interest in regard to this article. Both Gary D. Norsworthy and Diane D. Addie have received honoraria for speaking engagements and development of educational material, without conflicts of interest in regard to this paper. The other authors declare no conflicts of interest.

## Supporting information


**Table S1.** The breeds of purebred cats presented for chronic diarrhea and their FCoV infection status. This table shows a breakdown of the breeds of the 117 purebred cats, showing that 61/117 (52.1%) were under 1 year old; 77/117 (65.8%) were positive for FCoV RNA; that 47/77 (61.0%) of FCoV positive cats were under 1 year old and that 47/61 (71.2%) of cats under 1 year of age were FCoV positive.

## Endnotes

^1^
IDEXX Veterinary Diagnostic Laboratory, Westbrook, Maine, USA.

## References

[1] S. Marsilio , V. Freiche , E. Johnson , et al., “ACVIM Consensus Statement Guidelines on Diagnosing and Distinguishing Low‐Grade Neoplastic From Inflammatory Lymphocytic Chronic Enteropathies in Cats,” Journal of Veterinary Internal Medicine 37, no. 3 (2023): 794–816, 10.1111/jvim.16690. 37130034 PMC10229359

[2] C. H. Sung , S. Marsilio , B. Chow , et al., “Dysbiosis Index to Evaluate the Fecal Microbiota in Healthy Cats and Cats With Chronic Enteropathies,” Journal of Feline Medicine and Surgery 24 (2022): e1–e12, 10.1177/1098612X221077876. 35266809 PMC9160961

[3] D. G. O'Neill , D. Gunn‐Moore , S. Sorrell , et al., “Commonly Diagnosed Disorders in Domestic Cats in the UK and Their Associations With Sex and Age,” Journal of Feline Medicine and Surgery 25, no. 2 (2023): 1098612X231155016, 10.1177/1098612X231155016. PMC10812063 36852509

[4] E. M. Lund , P. J. Armstrong , C. A. Kirk , et al., “Health Status and Population Characteristics of Dogs and Cats Examined at Private Veterinary Practices in the United States,” Journal of the American Veterinary Medical Association 214, no. 9 (1999): 1336–1341, 10.2460/javma.1999.214.09.1336. 10319174

[5] G. D. Norsworthy , J. S. Estep , C. Hollinger , et al., “Prevalence and Underlying Causes of Histologic Abnormalities in Cats Suspected to Have Chronic Small Bowel Disease: 300 Cases (2008–2013),” Journal of the American Veterinary Medical Association 247, no. 6 (2015): 629–635, 10.2460/javma.247.6.629. 26331421

[6] A. E. Jergens , J. M. Crandell , R. Evans , M. Ackermann , K. G. Miles , and C. Wang , “A Clinical Index for Disease Activity in Cats With Chronic Enteropathy,” Journal of Veterinary Internal Medicine 24, no. 5 (2010): 1027–1033, 10.1111/j.1939-1676.2010.0549.x. 20584141

[7] A. E. Jergens , “Feline Idiopathic Inflammatory Bowel Disease: What We Know and What Remains to Be Unraveled,” Journal of Feline Medicine and Surgery 14 (2012): 445–458, 10.1177/1098612X12451548. 22736679 PMC10822384

[8] Y. I. Oh , K. W. Seo , D. H. Kim , and D. S. Cheon , “Prevalence, Co‐Infection and Seasonality of Fecal Enteropathogens From Diarrheic Cats in the Republic of Korea (2016–2019): A Retrospective Study,” BMC Veterinary Research 17 (2021): 367, 10.1186/s12917-021-03075-6. 34852811 PMC8633091

[9] N. C. Pedersen , J. F. Boyle , K. Floyd , et al., “An Enteric Coronavirus Infection of Cats and Its Relationship to Feline Infectious Peritonitis,” American Journal of Veterinary Research 42 (1981): 368–376. 6267960

[10] D. D. Addie and O. Jarrett , “A Study of Naturally Occurring Feline Coronavirus Infection in Kittens,” Veterinary Record 130 (1992): 133–137. 1313617 10.1136/vr.130.7.133

[11] A. Kipar , J. Kremendahl , D. D. Addie , W. Leukert , C. K. Grant , and M. Reinacher , “Fatal Enteritis Associated With Coronavirus Infection in Cats,” Journal of Comparative Pathology 119 (1998): 1–14. 9717123 10.1016/S0021-9975(98)80067-4 PMC7130287

[12] D. D. Addie and J. O. Jarrett , “Use of a Reverse‐Transcriptase Polymerase Chain Reaction for Monitoring Feline Coronavirus Shedding by Healthy Cats,” Veterinary Record 148 (2001): 649–653. 11400984 10.1136/vr.148.21.649

[13] S. J. Sabshin , J. K. Levy , T. Tupler , et al., “Enteropathogens Identified in Cats Entering a Florida Animal Shelter With Normal Feces or Diarrhea,” Journal of the American Veterinary Medical Association 241, no. 3 (2012): 331–337, 10.2460/javma.241.3.331. 22812469

[14] J. K. Paris , S. Wills , H.‐J. Balzer , et al., “Enteropathogen Co‐Infection in UK Cats With Diarrhoea,” BMC Veterinary Research 10, no. 13 (2014): 13, 10.1186/1746-6148-10-13. 24410914 PMC3896830

[15] A. Paul and J. Stayt , “The Intestinal Microbiome in Dogs and Cats With Diarrhoea as Detected by a Faecal Polymerase Chain Reaction‐Based Panel in Perth, Western Australia,” Australian Veterinary Journal 97, no. 10 (2019): 418–421, 10.1111/avj.12867. 31556108 PMC7159723

[16] S. Felten , U. Klein‐Richers , S. Unterer , et al., “Role of Feline Coronavirus as Contributor to Diarrhea in Cats From Breeding Catteries,” Viruses 14, no. 5 (2022): 858, 10.3390/v14050858. 35632600 PMC9143444

[17] K. Garraway , C. M. Johannes , A. Bryan , et al., “Relationship of the Mucosal Microbiota to Gastrointestinal Inflammation and Small Cell Intestinal Lymphoma in Cats,” Journal of Veterinary Internal Medicine 32, no. 5 (2018): 1692–1702, 10.1111/jvim.15291. 30084202 PMC6189339

[18] J. B. Honneffer , Y. Minamoto , and J. S. Suchodolski , “Microbiota Alterations in Acute and Chronic Gastrointestinal Inflammation of Cats and Dogs,” World Journal of Gastroenterology 20, no. 44 (2014): 16489–16497. 25469017 10.3748/wjg.v20.i44.16489 PMC4248192

[19] K. C. Polak , J. K. Levy , P. C. Crawford , C. M. Leutenegger , and K. A. Moriello , “Infectious Diseases in Large‐Scale Cat Hoarding Investigations,” Veterinary Journal 201, no. 2 (2014): 189–195, 10.1016/j.tvjl.2014.05.020. 24934262 PMC7110739

[20] T. A. Cave , M. C. Golder , J. Simpson , and D. D. Addie , “Risk Factors for Feline Coronavirus Seropositivity in Cats Relinquished to a UK Rescue Charity,” Journal of Feline Medicine and Surgery 6, no. 2 (2004): 53–58. 15123148 10.1016/j.jfms.2004.01.003 PMC7129206

[21] H. W. Chang , R. J. de Groot , H. F. Egberink , et al., “Feline Infectious Peritonitis; Insights Into Feline Coronavirus Pathobiogenesis and Epidemiology Based on Genetic Analysis of the Viral 3c Gene,” Journal of General Virology 91, no. Pt 2 (2010): 415–420. 19889934 10.1099/vir.0.016485-0

[22] S. Le Poder , A. L. Pham‐Hung d'Alexandry d'Orangiani , L. Duarte , et al., “Infection of Cats With Atypical Feline Coronaviruses Harbouring a Truncated Form of the Canine Type I Non‐Structural ORF3 Gene,” Infection, Genetics and Evolution 20 (2013): 488–494, 10.1016/j.meegid.2013.09.024. PMC7106123 24121017

[23] D. D. Addie , F. Bellini , J. Covell‐Ritchie , et al., “Stopping Feline Coronavirus Shedding Prevented Feline Infectious Peritonitis,” Viruses 15 (2023): 818, 10.3390/v15040818. 37112799 PMC10146023

[24] D. D. Addie , I. A. T. Schaap , L. Nicolson , and O. Jarrett , “Persistence and Transmission of Natural Type I Feline Coronavirus Infection,” Journal of General Virology 84 (2003): 2735–2744. 13679608 10.1099/vir.0.19129-0

[25] C. Dye , C. R. Helps , and S. G. Siddell , “Evaluation of Real‐Time RT‐PCR for the Quantification of FCoV Shedding in the Faeces of Domestic Cats,” Journal of Feline Medicine and Surgery 10, no. 2 (2008): 167–174. 18243744 10.1016/j.jfms.2007.10.010 PMC2582154

[26] M. Uiterwijk , R. Nijsse , F. N. J. Kooyman , et al., “Comparing Four Diagnostic Tests for Giardia Duodenalis in Dogs Using Latent Class Analysis,” Parasites and Vectors 11 (2018): 439, 10.1186/s13071-018-3014-2. 30064472 PMC6069568

[27] S. Meazzi , A. Stranieri , S. Lauzi , et al., “Feline Gut Microbiota Composition in Association With Feline Coronavirus Infection: A Pilot Study,” Research in Veterinary Science 125 (2019): 272–278. 31326703 10.1016/j.rvsc.2019.07.003 PMC7111766

[28] A. A. P. M. Herrewegh , M. Mahler , H. J. Hedrich , et al., “Persistence and Evolution of Feline Coronavirus in a Closed Cat‐Breeding Colony,” Virology 234 (1997): 349–363. 9268167 10.1006/viro.1997.8663 PMC7130968

[29] B. L. Haagmans , H. F. Egberink , and M. C. Horzinek , “Apoptosis and T‐Cell Depletion During Feline Infectious Peritonitis,” Journal of Virology 70, no. 12 (1996): 8977–8983. 8971027 10.1128/jvi.70.12.8977-8983.1996 PMC190995

[30] T. Takano , T. Hohdatsu , Y. Hashida , Y. Kaneko , M. Tanabe , and H. Koyama , “A “Possible” Involvement of TNF‐Alpha in Apoptosis Induction in Peripheral Blood Lymphocytes of Cats With Feline Infectious Peritonitis,” Veterinary Microbiology 119, no. 2–4 (2007): 121–131. 17046178 10.1016/j.vetmic.2006.08.033 PMC7117258

