# Supplementary material for: Feline Coronavirus Prevalence in 493 Cats With Chronic Diarrhea
Source: J Vet Intern Med. 2025 Apr 26;39(3):e70103. doi: 10.1111/jvim.70103 (PMC12033022; doi:10.1111/jvim.70103)
Supplement: Supplementary file 1 — Table S1. The breeds of purebred cats presented for chronic diarrhea and their FCoV infection status. This table shows a breakdown of the breeds of the 117 purebred cats, showing that 61/117 (52.1%) were under 1 year old; 77/117 (65.8%) were positive for FCoV RNA; that 47/77 (61.0%) of FCoV positive cats were under 1 year old and that 47/61 (71.2%) of cats under 1 year of age were FCoV positive. [file JVIM-39-e70103-s001.docx]

**Supplementary Table 1. The breeds of purebred cats presented for chronic diarrhea and their FCoV infection status.**

| **Breed** | **Number of cats** | **Number <1yo** | **Positive FCoV** | **FCoV positive <1yo** |
| --- | --- | --- | --- | --- |
| Abyssinian | 4 | 1 | 3 | 1 |
| American shorthair | 6 | 3 | 3 | 3 |
| Balinese | 1 | 0 | 1 | 0 |
| Bengal | 12 | 9 | 10 | 9 |
| Birman | 2 | 1 | 2 | 1 |
| Colourpoint shorthair | 1 | 0 | 1 | 0 |
| Cornish Rex | 2 | 0 | 2 | 0 |
| Devon Rex | 4 | 3 | 3 | 2 |
| Exotic shorthair | 3 | 1 | 2 | 1 |
| Himalayan | 5 | 2 | 2 | 1 |
| Japanese bobtail | 1 | 0 | 0 | 0 |
| Korat | 1 | 0 | 1 | 0 |
| Maine Coon | 9 | 8 | 8 | 8 |
| Munchkin | 1 | 1 | 1 | 1 |
| Persian | 11 | 3 | 7 | 2 |
| Ragdoll | 10 | 6 | 6 | 5 |
| Russian blue | 3 | 1 | 2 | 0 |
| Russian blue (X) | 2 | 1 | 1 | 1 |
| Savannah | 1 | 1 | 1 | 1 |
| Scottish Fold | 2 | 0 | 1 | 0 |
| Siamese | 15 | 10 | 10 | 6 |
| Siamese X | 12 | 5 | 4 | 1 |
| Siberian | 3 | 3 | 2 | 2 |
| Somali | 1 | 0 | 1 | 0 |
| Sphynx | 4 | 2 | 3 | 2 |
| Turkish | 1 | 0 | 0 | 0 |
| **TOTAL** | **117** | **61** (52.1%) | 77 (65.8%) | 47(61.0%) |

This table shows a breakdown of the breeds of the 117 purebred cats, showing that 61/117 (52.1%) were under one year old; 77/117 (65.8%) were positive for FCoV RNA; that 47/77 (61.0%) of FCoV positive cats were under 1 year old and that 47/61 (71.2%) of cats under one year of age were FCoV positive.
